# Expression of Somatostatin Receptor Subtypes (SSTR-1–SSTR-5) in Pediatric Hematological and Oncological Disorders

**DOI:** 10.3390/molecules25235775

**Published:** 2020-12-07

**Authors:** Kristof Harda, Zsuzsanna Szabo, Eva Juhasz, Balazs Dezso, Csongor Kiss, Andrew V. Schally, Gabor Halmos

**Affiliations:** 1 Department of Biopharmacy, Faculty of Pharmacy, University of Debrecen, 4032 Debrecen, Hungary; harda.kristof@pharm.unideb.hu (K.H.); szabo.zsuzsanna@pharm.unideb.hu (Z.S.); 2 Doctoral School of Pharmaceutical Sciences, University of Debrecen, 4032 Debrecen, Hungary; 3 Department of Pediatrics, Faculty of Medicine, University of Debrecen, 4032 Debrecen, Hungary; juhasze@med.unideb.hu (E.J.); kisscs@med.unideb.hu (C.K.); 4 Department of Pathology, Faculty of Medicine, University of Debrecen, 4032 Debrecen, Hungary; bdezso@med.unideb.hu; 5 Veterans Affairs Medical Center, Endocrine, Polypeptide and Cancer Institute, Miami, FL 33125, USA; Andrew.Schally@va.gov; 6 Department of Pathology, Miller School of Medicine, University of Miami, Miami, FL 33101, USA; 7 Department of Medicine, Divisions of Hematology-Oncology and Endocrinology, Miller School of Medicine, University of Miami, Miami, FL 33101, USA; 8 Sylvester Comprehensive Cancer Center, University of Miami, Miami, FL 33136, USA

**Keywords:** hematological-oncological disorders in children, somatostatin receptors, somatostatin analogs

## Abstract

Hematological and oncological disorders represent leading causes of childhood mortality. Neuropeptide somatostatin (SST) has been previously demonstrated in various pediatric tumors, but limited information exists on the expression and characteristics of SST receptors (SSTR) in hematological and oncological disorders of children. We aimed to investigate the expression of mRNA for SSTR subtypes (SSTR-1–5) in 15 pediatric hematological/oncological specimens by RT-PCR. The presence and binding characteristics of SSTRs were further studies by ligand competition assay. Our results show that the pediatric tumor samples highly expressed mRNA for the five SSTR subtypes with various patterns. The mRNA for SSTR-2 was detected in all specimens independently of their histological type. A Hodgkin lymphoma sample co-expressed mRNA for all five SSTR subtypes. SSTR-3 and SSTR-5 were detected only in malignant specimens, such as rhabdomyosarcoma, Hodgkin lymphoma, acute lymphoblastic leukemia, and a single nonmalignant condition, hereditary spherocytosis. The incidence of SSTR-1 and SSTR-4 was similar (60%) in the 15 specimens investigated. Radioligand binding studies demonstrated the presence of specific SSTRs and high affinity binding of SST analogs in pediatric solid tumors investigated. The high incidence of SSTRs in hematological and oncological disorders in children supports the merit of further investigation of SSTRs as molecular targets for diagnosis and therapy.

## 1. Introduction

Somatostatin is a widely distributed inhibitory peptide hormone that is involved in digestive, endocrine, and immune functions. Somatostatin acts primarily as an inhibitor of cell proliferation and hormone secretion with endocrine, paracrine, and autocrine activities. Somatostatin receptors (SSTR) are membrane-bound G-proteins that mediate physiological functions of somatostatin in the body [1]. Overexpression of SSTR has been found in pituitary adenomas (PA) and many human malignancies including lung, breast, and prostate cancers, colon carcinoma, glioblastoma, and uveal melanoma [2,3,4,5,6,7,8,9,10,11]. SSTR are divided into five basic subtypes: SSTR-1 (SSTR-1 gene location 14q13), SSTR-2 (17q24), SSTR-3 (22q13.1), SSTR-4 (20p11.2), and SSTR-5 (16p13.3). The antiproliferative action of SST and high expression of their corresponding SSTRs on various endocrine tumors led to the clinical application of synthetic stable analogs of SST for hormonal treatment of human malignancies such as acromegaly [2,4,7]. Based on structural similarity and reactivity for octapeptide and hexapeptide SST analogs, the receptor family can be divided into two subclasses: SSTR-2,3,5 react with SST octapeptide analogs and constitute members of one subgroup; SSTR-1,4 react poorly with these compounds and fall into another subgroup. [2,4,7,10,11,12,13,14,15]. Octapeptide analogs, such as octreotide (Sandostatin^®^), somatuline (Lanreotide^®^), and octastatin (Vapreotide^®^) are very popular drugs and they show high affinity to SSTR-2 and SSTR-5, moderate affinity to SSTR-3 and SSTR-4, and low binding affinity to SSTR-1. It is reported that octreotide has the highest affinity to SSTR-2, then, approximately similar affinity to SSTR-5 and SSTR-3, following lower binding affinity to SSTR-1, and displaying the lowest to SSTR-4. However, Lanreotide shows the highest affinity to SSTR-2 similarly to SSTR-5 and somehow lower binding affinity to SSTR-3 and SSTR-4, and displays the lowest affinity to SSTR-1. [12,13,14,15] Another novel SST analog pasireotide (SOM 230) possesses an affinity to SSTR-1, SSTR-3, and SSTR-5 [1]. In addition, synthetic SST analogs found other useful clinical applications as carrier molecules for radionuclides for tumor visualization and targeted radio- and chemotherapy of SSTR-positive tumors [2,4,7].

The high expression of SST-14 and somatostatin receptors in childhood neuroblastoma and medulloblastoma samples, as well as the clinical applicability of somatostatin receptor scintigraphy and radioreceptor-guided surgery have already been described [12,13,14,15,16]. Pediatric hematological and oncological diseases represent the second most frequent cause of childhood mortality after accidents, consequently, there is an urgent need for improved methods for early detection and development of novel treatment options of these disorders. Although the presence of SST has previously been demonstrated in certain pediatric tumors [17], only very limited information exists about the expression and characteristics of SSTRs in pediatric hematological and oncological disorders including malignant and benign conditions.

Fibrous dysplasia (FD) of bone is a genetic, non-inherited, rare benign bone disease existing in monostotic and polyostotic forms. FD is a developmental disease of bone in which there is replacement of normal spongiosa and filling of the medullary cavity of affected bones by abnormal fibrous tissue that contains trabeculae of poorly calcified primitive bone formed by osseous metaplasia. Infantile myofibromatosis (IM) is a mesenchymal disorder described by fibrous proliferation of the skin, bone, muscle, and viscera [18]. Although rare, it is the most common fibrous tumor in childhood [18,19,20]. Aggressive pediatric myofibromatosis is an autosomal recessive disease characterized by fibroblastic proliferation from cells derived from muscle-aponeurotic tissue. Its etiology is unknown, and the mean age of the reported cases is 7 years. The tumor shows rapid growth without serious pain and appears to be attached to the muscle tissue and/or bone. The treatment option is typically conservative surgical excision; however, early relapses have been reported [21]. Teratomas are special tumors with various cellular components that contain mature (i.e., benign) or immature (malignant) proliferating pluripotent cells of more than one germ-cell layers in origin. The incidence of teratomas is 1:4000 births [22]. The etiology of teratomas is not fully understood; however, they are likely to occur in part when individual pluripotent cells do not complete migration and continue dividing in an abnormal location, typically along the midline [23]. Regarding their nature, teratomas can be both benign and neoplastic. Mesenchymal hamartoma is a tumor-like benign mass of disorganized tissues reflecting a local developmental malformation with a poorly understood pathogenesis. It is uncommon in older children, especially after 2 years of age. The diagnosis of this tumor is difficult because the signs and clinical symptoms may be nonspecific; therefore, a high index of suspicion is required for diagnosis and treatment. Rhabdomyosarcoma (RMS) is the most common soft tissue sarcoma among children, with an annual incidence of 4.5 cases per 1 million children and is the third most frequent extracranial solid tumor of childhood after neuroblastoma and Wilms tumor [24]. RMS tumors are typically associated with the skeletal muscle lineage, and about 50% of cases are diagnosed in the first decade of life. Acute lymphoblastic leukemia (ALL) is the most frequent hematological neoplastic disease in children, characterized by the proliferation of transformed lymphoid cells in the bone marrow, peripheral blood, and other organs. The age-adjusted incidence rate of ALL in the United States is 1.38/100,000 individuals per year [25,26] with an estimated 5930 new cases and 1500 deaths in 2019 [26]. ALL accounts for 75–80% of acute leukemias in children [27,28]. Hodgkin lymphoma (HL) is a highly curable form of childhood cancer, with estimated 5-year survival rates exceeding 98% after treatment with chemotherapy alone or combined with radiotherapy (RT). Hereditary spherocytosis (HS) is the most frequent congenital red blood cell membrane defect in which abnormalities of red blood cell structural proteins lead to loss of erythrocyte membrane surface area, resulting in spherically shaped, hyperdense, poorly deformable red blood cells accompanied by hemolysis. Incidence of HS is 1/5000 among individuals of European descent and in the United States. Immune thrombocytopenia (ITP) is one of the most common bleeding disorders during childhood, occurring in ~5 to 10 per 100,000 children per year [27]. ITP in childhood is often a self-limited condition. Granulocytopenia is a heterogenous group of disorders accompanied by a decrease in peripheral blood granulocytes below the lower limit of normal range (<0.5 × 10^9^/l) [29,30]. Chronic benign neutropenia is the most common neutropenia in childhood. Neutropenia can be considered chronic if it does not improve after 8 weeks. In most children, neutropenia may persist for a few years and then resolve spontaneously. Children with chronic benign neutropenia are generally healthy and have a normal physical exam.

In the current study, we aimed to investigate the expression of mRNA for somatostatin receptor subtypes in a cohort of pediatric hematological and oncological specimens using RT-PCR and the binding characteristics of SSTR protein by ligand competition assays. In addition, we analyzed the correlation among the expression pattern of SSTR subtypes and clinicopathological characteristics of the pediatric patients. To the best of our knowledge, this is the first report showing the expression of SSTRs in various childhood tumors and hematological disorders. Our results may open up a new avenue for potential diagnostic and therapeutic applications of somatostatin analogs in pediatric oncology.

## 2. Results

### 2.1. Expression of SSTRs in Human Pediatric Solid Tumors and Hematological Diseases

**Figure 1:**
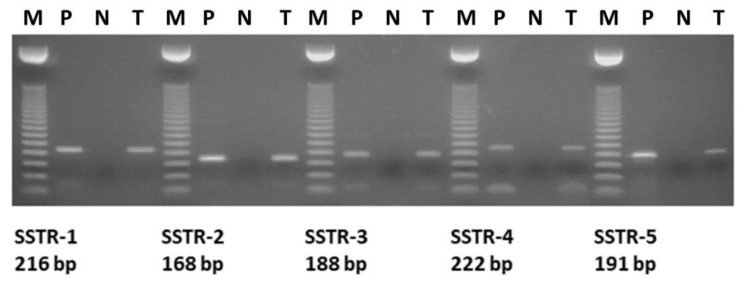
Representative RT-PCR analysis of mRNA for somatostatin receptor (SSTR) subtypes (SSTR-1–5) in one representative pediatric Hodgkin lymphoma specimen. M: DNA marker 50 bp; P: Positive control (human bladder carcinoma); N: Negative template control; T: Pediatric tumor specimen (patient number 4, Hodgkin lymphoma) positive for all 5 SSTR subtypes (SSTR-1–5).

**Figure 2:**
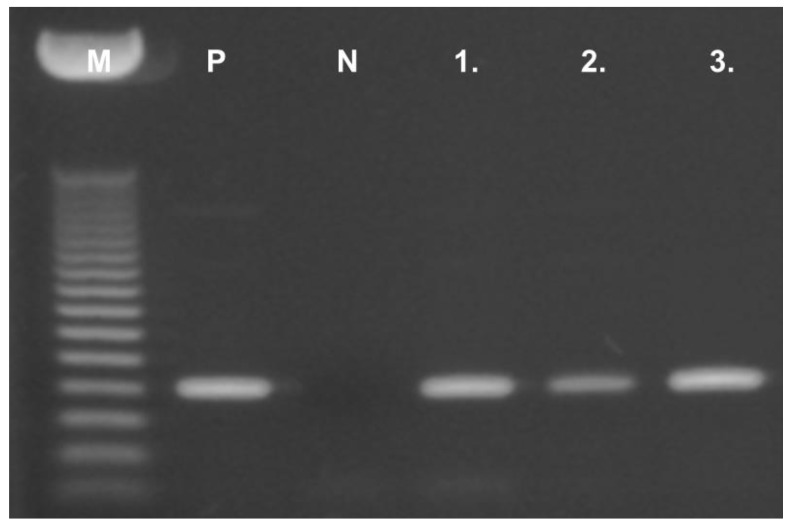
A representative figure of the expression of β-actin housekeeping gene in pediatric hematological and oncological specimens. M: DNA marker (50 bp); P: positive control (human pituitary); N: negative template control; 1–3: Representative pediatric samples.

We performed subtype-specific RT-PCR analyses on 15 pediatric hematological and oncological samples to investigate the mRNA expression of SSTR subtypes. The expected size of PCR products amplified with gene-specific primers was 216 bp for SSTR-1, 168 bp for SSTR-2, 188 bp for SSTR-3, 222 bp for SSTR-4, and 191 bp for SSTR-5, respectively (Figure 1). The mRNA expression pattern of the five SSTR subtypes examined, as well as the receptor binding characteristics of solid tumors and sample types for the human specimens are shown in Table 1. We found that the pediatric samples investigated highly expressed mRNA for the five subtypes of somatostatin receptors. The mRNA expression of SSTR-2 subtype was detected in all (15/15) of the samples independently from their histological type. Of the 15 specimens, only a HL sample expressed mRNA for all five SSTR subtypes and IM, FD, and ITP showed only one receptor subtype, SSTR-2. The expression of SSTR-5 was detected in two malignant solid tumor specimens (case 4, HL, and case 5, RMS). Of the 15 pediatric hematological and oncological specimens, PCR products for SSTR-3 could be detected only in malignant samples, two solid tumors (HL and RMS) and two cases (ALL and HS) expressed SSTR-3. Subtypes SSTR-3 and SSTR-5 were expressed in lower number of the specimens, found only in four and two samples, respectively. The incidence of SSTR-1 and SSTR-4 was similar (9/15, 60%) in the 15 specimens analyzed. Among all nine SSTR-4-positive samples, two were rhabdomyosarcomas, two specimens were ALL, one was HL, one was teratoma type, one histologically was defined as hamartoma, one as HS, and one was chronic benign neutropenia. Both RMS samples in our study mostly showed positivity for SSTR-1, SSTR-2, and SSTR-4. Both solid benign tumor samples showed SSTR-2 receptor expression, but none of them expressed SSTR-3 and SSTR-5 (Table 1). Template-free and reverse transcriptase-free controls excluded nonspecific amplification and DNA contamination. PCR amplification with specific primers for β-actin produced a single product in every sample, confirming no RNA degradation in the samples. As a positive control, human pituitary samples were used. (Figure 2)

### 2.2. Radioligand Binding Studies

**Figure 3:**
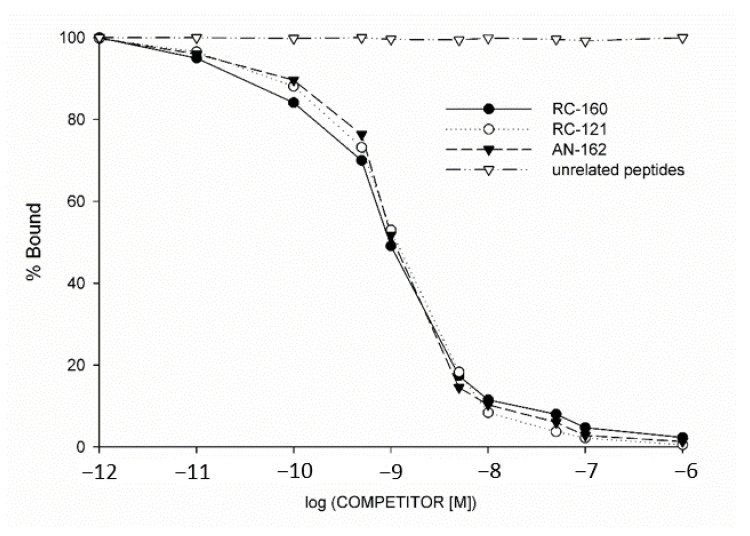
Representative displacement of [^125^I]RC-160 binding to membrane fractions of human rhabdomyosarcoma specimen by increasing concentrations of somatostatin (SST) analog RC-160 (Vapreotide) (●), cytotoxic SST analog AN-162 (▼) and RC-121, carrier peptide of AN-162 (○). Other unrelated peptides like LHRH, GHRH, VIP, bombesin, and epidermal growth factor (∇) did not displace the radioligand. Each point represents mean of duplicate or triplicate determinations.

The presence of SSTR protein, characteristics of these SST-binding sites and specific binding of radioiodinated SST analog RC-160 to membrane homogenates of human pediatric solid tumor samples were determined using ligand competition assays. Of the seven specimens examined, five malignant tumors and two benign tumor samples displayed SSTR binding (Table 1). Receptor binding affinities and concentrations of SSTRs in tumor membranes were also studied. Analyses of the typical displacement of [^125^I]RC-160 and the Scatchard plots of the specific binding data showed that the one-site model could provide the best fit. Based on these receptor binding results, the presence of one class of high affinity SSTR in crude membranes derived from human pediatric samples was indicated. The computerized nonlinear curve-fitting program and the Scatchard plot analyses of the SST receptor binding data in seven receptor-positive tumor samples showed that the single class of SSTRs had a mean dissociation constant (K_d_) of 6.00 nM (range 4.02–8.12 nM), with a mean concentration of SSTRs (maximal binding capacity, B_max_) of 391.1 fmol/mg membrane protein (range 255.1–760.8 fmol/mg protein). Biochemical specifications and parameters crucial to characterize specific binding sites were also defined. Thus, the binding of [^125^I]RC-160 was detected to be specific reversible, temperature- and time-dependent, and linear with protein concentration in the human pediatric tumor specimens examined. The specificity of SST binding sites was also demonstrated in competitive binding assays. Various peptides structurally related or unrelated to SST were used in these studies. The binding of radioiodinated RC-160 was displaced completely by increasing concentrations (10*^−^*^12^–10*^−^*^6^ M) of SST-14, whereas none of the structurally and functionally different and unrelated peptides examined, such as epidermal growth factor (EGF), luteinizing hormone-releasing hormone (LHRH), growth hormone-releasing hormone (GHRH), [Tyr^4^]bombesin, and insulin-like growth factor I inhibited the binding of radiolabeled SST octapeptide analog RC-160 at concentrations as high as 1 µM (Figure 3).

The expression of mRNA for SSTR subtypes was accompanied by ligand binding in all pediatric tumor specimens examined. Comparative analysis of the results of radioreceptor assays and SSTR subtype mRNA studies revealed that the expression of SSTR-2 and/or SSTR-5 subtypes was 100% consistent with the presence of specific binding sites for [^125^I] RC-160 (Table 1.). The binding affinity of SST analogs and cytotoxic SST analog AN-162 to membrane receptors of human pediatric cancer cells (HL and RMS) expressing SSTRs was also investigated by ligand competition assay. Displacement of [^125^I] RC-160 as a radioligand by the unlabeled SST analogs as competitor was determined. Our results show that SST octapeptide analogs RC-160 (vapreotide) RC-121 (as carrier peptide of AN-162) and cytotoxic SST analog AN-162 could effectively bind to SSTRs at low nanomolar concentration (Table 2, Figure 3). Nevertheless, the cytotoxic SST conjugate AN-162 had only slightly lower binding affinity to specific SSTRs than the free peptide carrier RC-121 (Table 2, Figure 3). Our results demonstrated that the high binding affinity of the carrier peptide RC-121 to tumoral SSTRs was fully preserved in the targeted cytotoxic analog of SST, AN-162.

### 2.3. Patient Follow-Up

In the group of samples investigated, 5 children died within 5 years (2 patients with RMS, one patient with teratoma, and two with ALL). The male to female ratio in the group investigated was 2:1 (10 boys, 5 girls). In the solid tumor group, four patients were still alive after 5 years, three children died, and the male to female ratio was 1.33:1 (4 male and 3 female patients). In the hematological diseases group, six patients were still alive after five years, two children with ALL died within 5 years and the male to female ratio was 3:1 (6 male and 2 female patients) (Table 3). In our study, among the five deceased patients, only two children showed relapse (both were ALL patients) that caused the death of these patients.

We did not detect any significant correlation among somatostatin receptor expression pattern and clinical outcome or clinicopathological characteristics.

## 3. Discussion

Despite stunning improvements since the mid-1990s, hematological and oncological diseases are still among the leading causes of childhood mortality [31]. As compared to adult oncology, at this moment, targeted oncotherapies play a much less important role among children with cancer. Moreover, combined chemotherapy protocols cannot be intensified any further without an unacceptable increase in acute toxicity and late complications. Thus, there is an urgent need for improved methods of early detection, accurate follow-up, and development of novel, effective treatment approaches based on recently identified molecular targets.

Pediatric cancer is relatively rare, with less than 13,500 cases and approximately 1500 deaths per year among children aged 0–14 worldwide [31]. Leukemia is by far the most common neoplastic disorder, accounting for about 33% of childhood cancers, followed by brain tumors (25%), lymphomas (8%), neuroblastoma (7%), and other rare pediatric malignancies such as Wilms tumor, soft tissue and bone sarcomas, and germ-cell tumors with similarly low relative incidence [31]. Treatment of childhood cancer depends on the type (histology and, ever increasingly, genotype) and the stage of the disease. Common treatment approaches include chemotherapy, surgery, radiation therapy, and stem cell transplantation. Innovative treatment modalities such as immunotherapy and targeted small molecular drugs become more and more important elements of first-line treatment in pediatric oncology [26,31,32].

**Figure 4:**
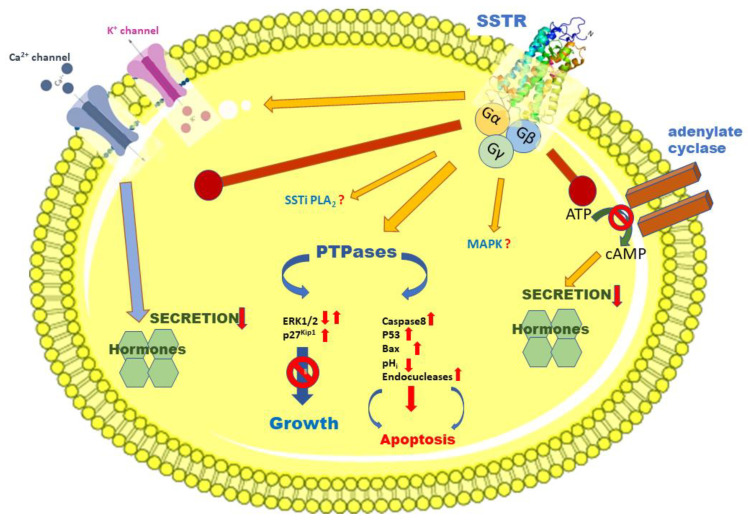
SSTR-based potential downstream pathways and signaling cascades leading to the modulation of hormone secretion, cell growth, and apoptosis. (based on references [10,11,12,15,37] with modifications).

Tumor-associated peptides provide attractive properties for treatment strategies due to easy access, convenient purification and storage. Furthermore, they are less immunogenic than antibody-based immunotherapies, have high tissue penetration and high affinity to specific cellular targets that influence cancer cell survival, proliferation, and differentiation [4,7,33]. They are characterized by a rapid clearance from the body and are prominent candidates for straightforward conjugation strategies [4,7,33]. Demonstration of the antiproliferative action of peptide hormone SST and the presence of its SSTRs on human endocrine tumors clearly led to the application of efficient and biologically stable synthetic octapeptide analogs such as RC-160 (octastatin/Vapreotide^®^), SMS-201-995 (ocreotide/Sandostatin^®^), SOM 230 (pasireotide), and BIM-23014 (somatuline/Lanreotide^®^) for hormonal treatment of these malignancies [2,4,7,12,16]. These SST analogs are widely used for the treatment of acromegaly, pancreatic neuroendocrine tumors, endocrine tumors of the gastroenterohepatic system, including carcinoid tumors, glucagonomas, gastrinomas, insulinomas, and VIPomas [2,4,7,12,16,34]. Based on our best knowledge, there are no definitive or conclusive data available in the literature showing the potential activation of other receptors by these SST octapeptide analogs. In addition, it has been demonstrated that SST analogs could be effectively used as carriers of radionuclides for visualization and targeted radio- or chemotherapy of SSTR-positive tumors [4,7,16,35]. Many tumor cells of various origins, such as well-differentiated gastroenteropancreatic neuroendocrine neoplasms as well as other cancers show overexpression of SSTR, which can serve as the molecular basis of targeted diagnostic and treatment methods [2,4,7,9,36]. The schematic SSTR-based potential downstream pathways and signaling cascades leading to the modulation of hormone secretion, cell growth, and apoptosis are shown in Figure 4.

Based on these findings, SST peptide analogs and SSTRs may have great diagnostic and therapeutic potential in pediatric hematology and oncology. Similarly to adult oncology, radiolabeled somatostatin analogs might offer an effective tool for identifying the localization and extent of tumors in children. Very limited data are available on somatostatin analogs in childhood tumors. Most of these data are not about malignancies, and almost all data were obtained with octreotide [38]. In addition, Dishop and Kuruvilla reported that SST analogs are also used therapeutically to reduce the symptoms and side-effects of chemotherapy and to induce differentiation in pediatric oncology [39]. It has not been established whether SSTRs could be used for the localization and treatment of hematological and oncological disorders in children and the previous findings available are incomplete and inconclusive. Therefore, in our present study, we aimed to investigate the expression of mRNA for SSTR subtypes (SSTR-1–5) in samples from human pediatric solid tumors as well as bone marrow aspirates and peripheral blood of children with hematological diseases. We also studied the binding characteristics of SSTR protein by radioreceptor assay. Moreover, we analyzed the potential correlation among the expression of the SSTRs, their binding characteristics, and clinicopathological data of the pediatric patients.

Our results show that the 15 pediatric samples investigated highly expressed mRNA for all five subtypes of SSTRs with various patterns. No sample was found without the expression of at least one of the SSTR subtypes. It is noteworthy that only a HL sample expressed mRNA for all five SSTR subtypes. Similarly, only SSTR-2 was observed in IM, FD, and ITP. Two malignant solid tumor specimens showed the expression of SSTR-5 and PCR products for SSTR-3 could be detected only in malignant samples. The incidence of SSTR-1 and SSTR-4 was similar (9/15, 60%) in the 15 specimens analyzed. Among the nine SSTR-4 positive samples, two were RMS, two ALL, one was HL, one teratoma-type, one histologically was defined as hamartoma, one as HS, and one as chronic benign neutropenia. Among the five ALL samples, only the sample of the female patient was positive for SSTR-3, and none of the four samples from male children expressed mRNA for SSTR-3. The very limited amount of the biological samples did not allow us to perform Western blot or immunohistochemistry. However, in seven cases we were able to prepare crude membrane protein fractions for radioligand binding studies to demonstrate the presence of specific SSTR binding sites. Radioligand binding studies also confirmed the presence of specific, high affinity SSTRs in pediatric solid tumors investigated with a mean dissociation constant (K_d_) of 6.00 nM and mean maximal binding capacity (B_max_) of 391.1 fmol/mg protein. Molecular biology analyses and radioligand binding studies clearly demonstrated that the expression of mRNA for SSTR-2 and SSTR-5 subtypes was 100% consistent with the presence of specific receptors for radiolabeled SST octapeptide analog RC-160.

Although the size of the investigated cohort is rather small and its composition is heterogenous, it is worth to note that in the examined group, five children with cancer died during the 5 year follow-up period. Three of the five ALL patients were alive, but two children died within 5 years. Patients diagnosed with rhabdomyosarcoma and teratoma showed the worst outcome in this cohort. Correlation between SSTR expression and clinical data was not observed. The high binding affinity of the synthetic octapeptide analogs to SSTR in malignant pediatric samples suggests that children with SSTR-positive cancers might be good candidates for therapy with SST analogs, including the targeted cytotoxic SST analog AN-162.

Our results demonstrated for the first time that somatostatin receptors are highly expressed in childhood benign and malignant solid tumors and in samples of pediatric hematological disorders. These findings might open up a new avenue for a potential application of peptide hormone analogs for the detection and treatment of pediatric oncological and hematological disorders. However, to clarify the therapeutic and clinical significance of SST receptors in pediatric oncology and hematology, further studies are needed. Our results may also help to better understand the exact mechanism of pediatric hematological and oncological disorders and provide better approaches for the early detection of these malignancies. Our findings may facilitate the potential clinical application of synthetic analogs of somatostatin or its radionuclide or cytotoxic derivatives for diagnostic or therapeutic purposes.

## 4. Materials and Methods

### 4.1. Human Samples

We examined 15 pediatric hematological and oncological specimens. All available clinicopathological data, including age at diagnosis, sex, tumor type or type of hematological disorder based on histopathologic examination, sample type, and survival, are shown in Table 1. Average age of patients was 8.03 years (range: 9 months–15 years). Human samples were collected from patients treated at the Department of Pediatric Hematology-Oncology, University of Debrecen, Hungary. Seven patients had solid tumors, eight children had malignant or benign type of hematological disorders. Seven samples were bone marrow aspirates, one hematological specimen was obtained from peripheral blood (Table 3). Solid tumor tissues were obtained at the time of primary surgery. All the samples were processed for routine histopathological examination and the pathological diagnosis was confirmed by a local pathologist. For molecular biology analysis, human bladder tumor tissue was used as an SSTR-positive control. Local Institutional Ethics Committee approved the collection and use of these specimens for the current study and informed consent was obtained. Tumor tissues were immediately frozen in liquid nitrogen and stored at −80 °C until further processing. All diagnostic interventions were performed based on suspected neoplastic conditions.

### 4.2. Histology

Histopathological examination of the 15 human pediatric hematological and oncological samples involved in our study revealed that five patients were suffering from ALL. Two samples were classified as RMS and one sample each was characterized histologically as teratoma, IM, FD, HL, hamartoma, ITP, HS, and chronic benign neutropenia (Table 3).

### 4.3. RNA Isolation

Homogenization of the samples was performed with Tissue Ruptor (IKA^®^-WERKE GmbH). Total RNA was isolated with an RNA Isolation Kit (Macherey-Nagel) according to the manufacturer’s protocol. Isolated total RNA was redissolved in RNAs-free water and quantity and quality were measured by a NanoDrop spectrophotometer (ND-1000, Thermo Fisher Scientific Inc.). Whenever enough material was available, experiments were performed in triplicate.

### 4.4. RT-PCR Analysis of Total RNA Samples

Total RNA was analyzed using a MMLV kit (Promega Co., Madison, WI, USA). The reverse transcriptase reaction mix (25 mM MgCl2; 10 × PCR buffer [500 mM KCl, 100 mM Tris-HCl, pH 8.3], distilled, autoclaved water, 1 mM dinucleotides, 2.5 U/µL MuLV reverse transcriptase, 1 U/µL RNase inhibitor, and 2.5 µM oligo dT) was added together with 200 ng RNA template. The total reaction volume of 20 µL was incubated at 23 °C for 10 min and 42 °C for 15 min, followed by 99 °C for 5 min.

RT-PCR reaction was performed in 25 μL reaction volume with gene-specific primers. The primers for PCR amplification were designed using the published sequences for the respective genes as shown in Table 4. Primer sequences with as small as possible homology among SST receptor subtypes were selected as described before [6,8].

For PCR reaction mixture 1 × PCR buffer, 1 U of Taq Polimerase (Invitrogen), 1.5 mM of MgCl_2_, 200 μM of dNTP (Fermentas), 0.5 µM of each of the gene specific primers (SSTR-1, SSTR-2, SSTR-3, SSTR-5), (Invitrogen), and 3 µL cDNS template was used in 25 µL reaction volume. For SSTR-4 RT-PCR reaction 300 µM dNTP (Fermentas) was added and 3 µL cDNS template was used in 25 µL volume as well.

PCR reaction was performed in a C1000 TM Thermal Cycler RT-PCR system (Bio-Rad Laboratories, Inc.). To run the RT-PCR, the following PCR protocol was used:

• for SSTR-1, SSTR-2, SSTR-4, SSTR-5: denaturation (3 min at 94 °C), 40 cycles (94 °C for 45 s, 60 °C for 30 s and 70 °C for 90 s);

• for SSTR-3: denaturation (3 min at 94 °C) was followed by 40 cycles (94 °C for 45 s, 63 °C for 30 s and 70 °C for 90 s).

The PCR reaction was finished by a prolonged extension time of 72 °C for 10 min.

PCR products were separated in a 1.5% agarose gel containing GelRed and detected under UV light, digitalized with AlphaDigiDoc™ RT (Alpha Innotech). To determine the size of the DNA, a 50 bp DNA marker (Bioline) was used. To test the quality of the RT-PCR, β-actin was used as a positive internal control for each of the transcribed RNA samples (Figure 2).

### 4.5. Radioligand Binding Studies

The radioiodinated derivatives of the RC-160 SST analogue were prepared by the chloramine-T method and purified by reverse phase high-performance liquid chromatography (RP-HPLC) as described previously [6]. Somatostatin receptor-binding assays were performed as described previously [6] with some minor modifications, using in vitro ligand competition assays based on the binding of [^125^I] RC-160 as a radioligand to membrane fractions of the pediatric samples. This radioiodinated ligand has been well characterized and reported previously and has high affinity for SSTR-2 and SSTR-5 [4]. Tumor membrane homogenates were incubated as competitors with 50,000–70,000 cpm radioiodinated RC-160 and 10^−12^–10^−6^ M nonradioactive peptides. After incubation for 2 h, the binding reactions were stopped, and the bound ligand was separated and counted in a gamma counter. The LIGAND-PC computer curve-fitting software of Munson and Rodbard was used to determine the type of receptor binding, the dissociation constant (K_d_), and the maximal binding capacity of SSTRs (B_max_) [6,8]. The binding potencies of SST analogs and cytotoxic SST analog AN-162 to SSTRs were also determined by displacement of [^125^I]-RC-160 binding. The final binding affinities were expressed as IC_50_ values. Protein concentration was determined by the method of Bradford using a Bio-Rad protein assay kit (Bio-Rad Laboratories, Inc.).

## Figures and Tables

**Table 1:** Expression of mRNA for somatostatin receptor subtypes (SSTR-1–5) and binding characteristic of somatostatin receptors in pediatric hematological and oncological specimens.

Patient Number	Sample Type	SSTR-1	SSTR-2	SSTR-3	SSTR-4	SSTR-5	SSTR	
							K_d_ (nM)	B_max_ (fmol/mg Protein)
1	Teratoma	-	+	-	+	-	5.76	274.0
2	Myofibromatosis	-	+	-	-	-	6.85	273.3
3	Fibrous dysplasia	-	+	-	-	-	4.94	255.1
4	Hodgkin lymphoma	+	+	+	+	+	8.01	760.8
5	Rhabdomyosarcoma	+	+	-	+	+	8.12	505.1
6	Rhabdomyosarcoma	+	+	+	+	-	4.31	371.4
7	Hamartoma	+	+	-	+	-	4.02	297.9
8	Acute lymphoblastic leukemia	+	+	+	-	-	N/A	
9	Acute lymphoblastic leukemia	+	+	-	-	-	N/A	
10	Acute lymphoblastic leukemia	+	+	-	-	-	N/A	
11	Acute lymphoblastic leukemia	-	+	-	+	-	N/A	
12	Acute lymphoblastic leukemia	-	+	-	+	-	N/A	
13	Immune thrombocytopenia	-	+	-	-	-	N/A	
14	Hereditary spherocytosis	+	+	+	+	-	N/A	
15	Chronic benign neutropenia	+	+	-	+	-	N/A	

K_d_ and B_max_ values were calculated from duplicates; K_d_: dissociation constant; B_max_: maximal binding capacity; N/A: not analyzed.

**Table 2:** Receptor binding potency (IC_50_ values) of SST analogs to the membrane receptors of human pediatric cancer cells.

Compound	IC_50_ Values (nM)	
	Hodgkin Lymphoma (Patient No.4)	Rhabdomyosarcoma (Patient No.5)
RC-160 (Vapreotide^®^)	0.86	0.73
RC-121	1.04	0.92
AN-162	1.53	1.19

IC_50_ values were calculated by computerized curve-fitting program from displacement experiments as described earlier [6,8]. IC_50_ is defined as the dose causing 50% inhibition of specific binding of [^125^I] RC-160 to the membranes. Values are means of two to three determinations.

**Table 3:** Clinicopathology data of 15 pediatric hematological and oncological specimens.

Sample	Histological Type	Gender	Sample Type	Age at Sample Collection	5-Year Survival
1	Teratoma/adenocarcinoma component	Male	Mediastinal tumor tissue	14.5 year	died
2	Juvenile myofibromatosis	Male	Right hip bone tumor tissue	2 year	alive
3	Fibrous dysplasia	Female	Bone tumor tissue	10.5 year	alive
4	Hodgkin lymphoma	Female	Lymph node	15 year	alive
5	Rhabdomyosarcoma	Male	Pelvic tumor tissue	12.5 year	died
6	Rhabdomyosarcoma	Female	Neck mass biopsy	8 year	died
7	Benign mesenchymal hamartoma	Male	Mediastinal mass	4 year	alive
8	Acute lymphoblastic leukemia	Female	Bone marrow	5 year	alive
9	Acute lymphoblastic leukemia	Male	Bone marrow	13 year	alive
10	Acute lymphoblastic leukemia	Male	Bone marrow	9.5 year	died
11	Acute lymphoblastic leukemia	Male	Bone marrow	6.5 year	alive
12	Acute lymphoblastic leukemia	Male	Peripheral blood	15 year	died
13	Immune thrombocytopenia	Male	Bone marrow	10 month	alive
14	Hereditary spherocytosis	Female	Bone marrow	9 month	alive
15	Chronic benign neutropenia	Male	Bone marrow	12.5 month	alive

**Table 4:** Sequences of SSTR-1, -2, -3, -4, -5 primers used for RT-PCR assay.

SSTR-1 sense	5′-TAT CTG CCT GTG CTA CGT GC-3’(1 exon)
SSTR-1 antisense	5’-GAT GAC CGA CAG CTG ACT CA-3′(1 exon)
SSTR-2 sense	5′-CGG AGT GAC AGT AAG CAG GA-3′(1 exon)
SSTR-2 antisense	5′-CGA AGC CAG TGT GGG TAGG-3′(1 exon)
SSTR-3 sense	5’-TGA GTC ACC AAC GTC TAC ATCC-3’(1 exon)
SSTR-3 antisense	5’-ACG CTC ATG ACA GTC AGG C-3’(1 exon)
SSTR-4 sense	5′-CGC TAC GCC AAG ATG AAG A-3′(1 exon)
SSTR-4 antisense	5′-AGA CAG AAG ACG CTG GTG AA-3′(1 exon)
SSTR-5 sense	5’-CGT CTT CAT CAT CTA CAC GG-3’(1 exon)
SSTR-5 antisense	5’-GGC CAG GTT GAC GAT GTT GA-3’(1 exon)
